# A RabGAP Regulates Life-Cycle Duration via Trimeric G-protein Cascades in *Dictyostelium discoideum*

**DOI:** 10.1371/journal.pone.0081811

**Published:** 2013-12-11

**Authors:** Hidekazu Kuwayama, Yukihiro Miyanaga, Hideko Urushihara, Masahiro Ueda

**Affiliations:** 1 Faculty of Life and Environmental Sciences, University of Tsukuba, Tsukuba, Japan; 2 Department of Biological Sciences, Graduate School of Science, Osaka University, Toyonaka, Japan; 3 Japan Science and Technology Agency, Core Research for Evolutional Science and Technology, Toyonaka, Japan; 4 Quantitative Biology Center, RIKEN, Osaka, Japan; Tohoku University, Japan

## Abstract

**Background:**

The life-cycle of cellular slime molds comprises chronobiologically regulated processes. During the growth phase, the amoeboid cells proliferate at a definite rate. Upon starvation, they synthesize cAMP as both first and second messengers in signalling pathways and form aggregates, migrating slugs, and fruiting bodies, consisting of spores and stalk cells, within 24 h. In *Dictyostelium discoideum*, because most growth-specific events cease during development, proliferative and heterochronic mutations are not considered to be interrelated and no genetic factor governing the entire life-cycle duration has ever been identified.

**Methodology/Principal Findings:**

Using yeast 2-hybrid library screening, we isolated a *Dictyostelium discoideum* RabGAP, Dd Rbg-3, as a candidate molecule by which the *Dictyostelium* Gα2 subunit directs its effects. Rab GTPase-activating protein, RabGAP, acts as a negative regulator of Rab small GTPases, which orchestrate the intracellular membrane trafficking involved in cell proliferation. Deletion mutants of *Dd rbg-3* exhibited an increased growth rate and a shortened developmental period, while an overexpression mutant demonstrated the opposite effects. We also show that Dd Rbg-3 interacts with 2 Gα subunits in an activity-dependent manner *in vitro*. Furthermore, both human and *Caenorhabditis elegans rbg-3* homologs complemented the *Dd rbg-3*–deletion phenotype in *D. discoideum*, indicating that similar pathways may be generally conserved in multicellular organisms.

**Conclusions/Significance:**

Our findings suggest that Dd Rbg-3 acts as a key element regulating the duration of *D. discoideum* life-span potentially via trimeric G-protein cascades.

## Introduction

Rab GTPase belongs to a member of the Ras GTPase superfamily and exerts specialized functions, including the control of intracellular endocytic and exocytic membrane trafficking and organelle biogenesis. The activity of Rab must be finely tuned to fulfill these complex cellular activities via GDP-GTP exchange and GTP hydrolysis. Rab GTPase-activating protein (RabGAP) facilitates the inactivation of Rab by accelerating GTP hydrolysis and is characterized by the Tre2-Bub2-Cdc16 (TBC) domain, which is responsible for binding to and regulation of Rab [1]. Recent studies have revealed that RabGAPs potentially regulate the signaling cascade among different types of Rabs or between Rabs and other small GTPases [1]. Thus, RabGAP is considered to play a negative role, not only in regulating cell proliferation, but also in various cellular events, such as the formation of primary cilia, macropinocytosis, immunological synapse formation, cytokinesis, and autophagy. Despite its functional importance, little is known about the signaling pathways that lead to the modulation of the activity of RabGAPs, especially within the context of dynamic higher dimensional biological processes, like morphogenesis and development.

Development comprises spatiotemporally regulated processes, including cell proliferation and differentiation. In a model organism, *Dictyostelium discoideum*, these two processes are distinctly separated at the proliferation and developmental phases during its life-cycle, respectively; therefore, this micro-organism is suitable for distinguishing between molecular functions involved in these biological events. During cell proliferation, these cells sense their food source using a chemosensory system and internalize food particles by phagocytosis or pinocytosis. Within 24 h after exhaustion of the food source, the cells go through a series of developmental stages, viz., aggregation, slug formation, and eventual formation of a fruiting body [2].

Cell aggregation is initiated by periodical secretion of extracellular adenosine 3′,5′-cyclic monophosphate (cAMP). Extracellular cAMP has been identified as a regulatory molecule that plays a central role, not only in chemotaxis, but also in cell differentiation and morphogenesis [3], [4]. Binding of extracellular cAMP to its specific cell-surface serpentine receptors results in the transient dissociation of a heterotrimeric G-protein into Gα2 and Gβγ subunits, which are anchored on the inside of plasma membrane. The activation of G-protein, in turn, evokes a signal transduction cascade that controls both chemotaxis by regulating actin cytoskeleton via parallel pathways, including phosphatidyl inositol signaling and cAMP production, by regulating adenylate cyclase [5]. It has been demonstrated that dominant mutations in the *gα2* gene results in partial, but significant, inhibition of adenylyl cyclase activation [6], [7]. Cells lacking Gα2 are neither able to respond toward cAMP by chemotaxis, nor activate adenylyl cyclase, and are thus not able to form any multicellular structures or differentiate. These observations indicate that Gα2 is tightly coupled to the developmental program in *D. discoideum*.

*Rbg-3* encodes a novel RabGAP containing a TBC domain [8], [9]. *Rbg-3* was first isolated as an interacting partner of TUB-1 in *Caenorhabditis elegans* [10]. Mutation of *tub-1* has been shown to have an effect on lifespan, in a manner that is dependent on daf-16/FOXO, as well as on fat deposition [11]. In *tub-1*-null phenotypes, silencing of *rbg-3* resulted only in decreasing fat deposition, suggesting that TUB-1 may directly regulate fat deposition via the Rbg-3 pathway [12].

In this study, we have attempted to establish the *in vivo* function of RabGAP by engineering deletion and overexpression mutants of *Dd rbg-3*. The deletion mutant showed both faster proliferation and development, while overexpression of the gene resulted in the opposite effects. Furthermore, since Dd Rbg-3 was isolated as a candidate binding partner of a dominant form of Gα2 by yeast 2-hybrid (Y2H) screen and was shown to co-immunoprecipitate with Gα2, the activity of Dd Rbg-3 is most likely regulated by a Gα2-dependent signaling pathway, via extracellular cAMP stimulation. In addition, Gα9, which is responsible for cell proliferation, was identified as another binding partner of Dd Rbg-3. Furthermore, expression of human and *C. elegans rbg-3* could rescue the deletion mutant, indicating that the Rbg-3 signaling pathway may also play a similar role in mammals and nematodes, with or without the tubby cascade. We further discuss the possible involvement of Dd Rbg-3 in the regulation of the duration of the full life-cycle via Gα signaling.

## Results

### Characterization of *Dictyostelium discoideum* Rbg-3

**Figure 1:**
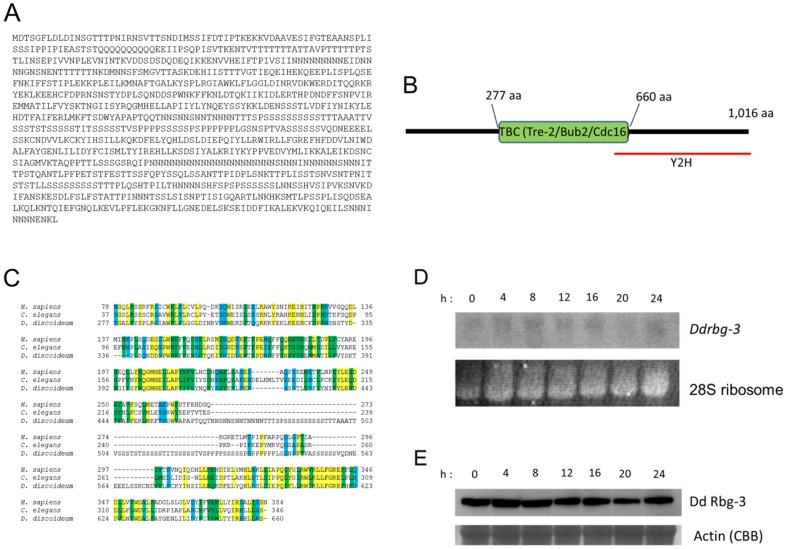
Sequence analyses and expression of *Dictyostelium discoideum Rbg-3* (*Dd Rbg-3*). (A) Deduced amino acid sequence of Dd Rbg-3. (B) Schematic diagram of the Dd Rbg-3 domain structure. (C) Multiple sequence alignment of the TBC domains of Rbg-3 homologs. Residues identical among the 3 proteins compared are indicated in yellow, conserved residues in green, and semi-conserved residues in blue. (D) Northern blot analysis of *Dd Rbg-3* during development. Ten micrograms of total RNA obtained from the organism at different developmental time-points were loaded in each gel lane, and the blots hybridized with an *Dd Rbg-3*-specific probe. (E) Western blot analysis of Dd Rbg-3 during development. One hundred micrograms of total protein, obtained from the organism at different developmental time-points, were loaded in each gel lane, and the blots probed with a Dd Rbg-3-specific antibody.

*Dd rbg-3* was identified in a Y2H screen in which the active form of Gα2 (Q208L) was utilized as bait [7]. The prey clone isolated in this way corresponded to a RabGAP (Fig. 1A; GenBank accession no. XM_635392.1) with a predicted TBC domain approximately in the middle of the protein, between amino acids 277 and 660 (Fig. 1B). However, the isolated *Dd rbg-3* cDNA fragment encoded a partial amino acid sequence representing the C-terminal domain [residues 523–1016] of the RabGAP (Fig. 1B; red line). Although the TBC domain of Dd Rbg-3 includes a long stretch of poly-serine and poly-phenylalanine, the remaining regions of the TBC domain are related to both human and *C. elegans* TBC domains, with 51.4% identity and 28.4% similarity at the amino acid level (Fig. 1C).

During the *Dictyostelium* life-cycle, *Dd rbg-3* showed a low level of mRNA expression in the vegetative stage; this increased marginally after 8-h starvation (aggregation stage), decreased again moderately for up to 20 h [slug stage], after which it again increased for up to 24 h (Fig. 1D). In contrast, the expression of the Dd Rbg-3 protein appeared to be constant throughout the life-cycle (Fig. 1E), except for a slight decrease at 16 h (culmination stage). These results suggest potential functions for Dd Rbg-3 during both growth and development.

### *Dd rbg-3* mutants demonstrate altered life-cycle duration

*Dd rbg-3* gene disruption was performed using homologous recombination in an axenic strain AX2 as wild-type (WT). Three independent *Dd rbg-*3–null mutants were isolated, which were phenotypically indistinguishable. Therefore, a single clone was selected for subsequent analyses. The null mutation was confirmed by Southern blot analysis (Fig. S1A and S1B). Concurrently, an overexpression mutant was made by transformation of AX2 with an actin15 promoter-driven extrachromosomal vector [13]. Northern blot analysis of the *Dd rbg-3*-null and *Dd rbg-3*-overexpression mutants revealed no detectable *Dd-rbg3* mRNA and high levels of *Dd rbg-3* mRNA, respectively, throughout the life-cycle (Fig. S1C).

**Figure 2:**
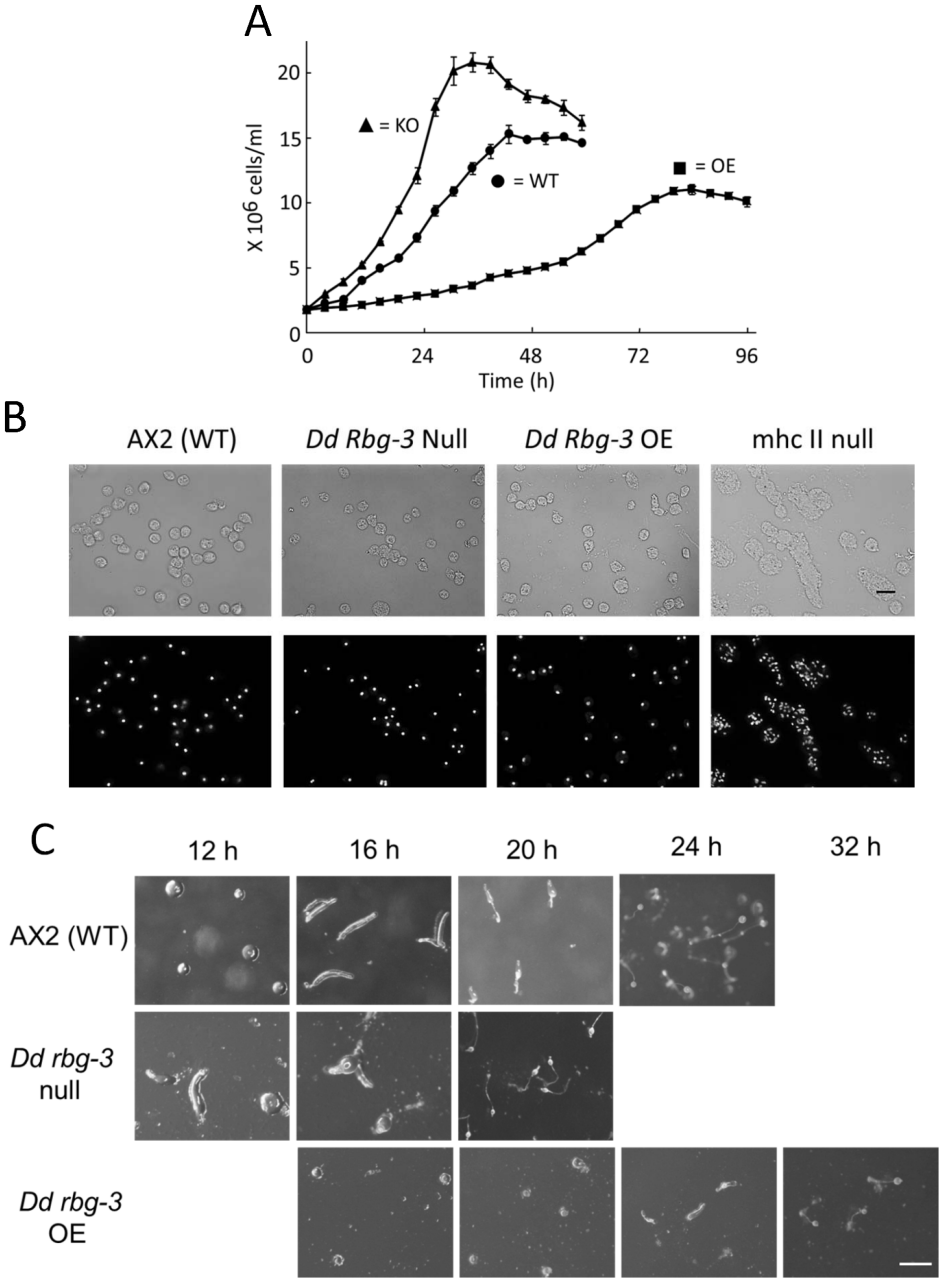
Phenotypes of *Dd rbg-3-*null and *Dd rbg-3-*overexpression mutant cells. (A) Cell growth in AX2 (filled circles, •), *Dd rbg-3–*null (filled triangles, ▴), and *Dd rbg-3*-overexpression (filled squares, ▪) strains. Cells were cultured in HL5 liquid medium, at 21°C, with shaking at 125 rpm. (B) Vegetative cell shape of AX2, *Dd rbg-3*-null, and *Dd rbg-3*-overexpression cells. To observe nuclei, vegetative cells were incubated with 0.1 µg/ml DAPI, after fixation with 1% formaldehyde in cold methanol, and 3 washes with PB. Scale bar, 20 µm. (C) Development in the parental AX2 strain, *Dd rbg-3*-null, and *Dd rbg-3*-overexpression cells were assessed on non-nutrient agar plates; cells were plated at a density of 1×10^6^ cells/cm^2^. In AX2 cells, mounds formed at 12 h after plating; slugs were visible at 16 h; culmination occurred by 20 h, and fruiting bodies were observed at 24 h. Development concluded faster in the *Dd rbg-3*-null strain, and slower in the *Dd rbg-3*-overexpression strain. Scale bar, 1 mm.

Growth of the mutant strains were examined under shaking-culture conditions in a standard *Dictyostelium* liquid nutrient, HL5 [14]. Cell numbers of the null mutant increased faster, and the maximum cell density at the stationary phase was higher, than that of the parental AX2 cells (Fig. 2A); in contrast, overexpression of *Dd rbg-3* resulted in markedly lower growth rate (Fig. 2A). However, there were no abnormalities in cell division, nuclear morphology, or cytokinesis in the mutant strains in comparison with such a mutant, *mhcII*–null (Fig. 2B). These results indicated that *Dd rbg-3* plays an inhibitory role during the cell cycle.

When the cells were starved on a non-nutrient agar surface, *Dd rbg-3*–null cells fulfilled development by 4 h faster than the wild-type parental strain, AX2 (Fig. 2C). By 12 h, the null mutant was already at the slug stage; it reached the culmination stage by 16 h and formed normal fruiting bodies by only 20 h after commencement of starvation culture. In contrast, AX2 cells constitutively overexpressing *Dd rbg-3* showed the opposite phenotype (Fig. 2C). Aggregation and mound formation only occurred 20 h after starvation culture commenced, while normal fruiting bodies were observed only after 32–36 h. However, there was no distinct difference in the expression of developmental genes between these 2 types of mutants (Fig. S2). These results indicated that the expression of Dd Rbg-3 does not determine cell differentiation and morphogenesis but retards the life-cycle duration of *D. discoideum*. Thus, we concluded that Dd Rbg-3 is a regulator in the life-cycle span of *D. discoideum*.

### Overexpression of Dd Rbg-3 increases the size of lysosomes during cell proliferation

**Figure 3:**
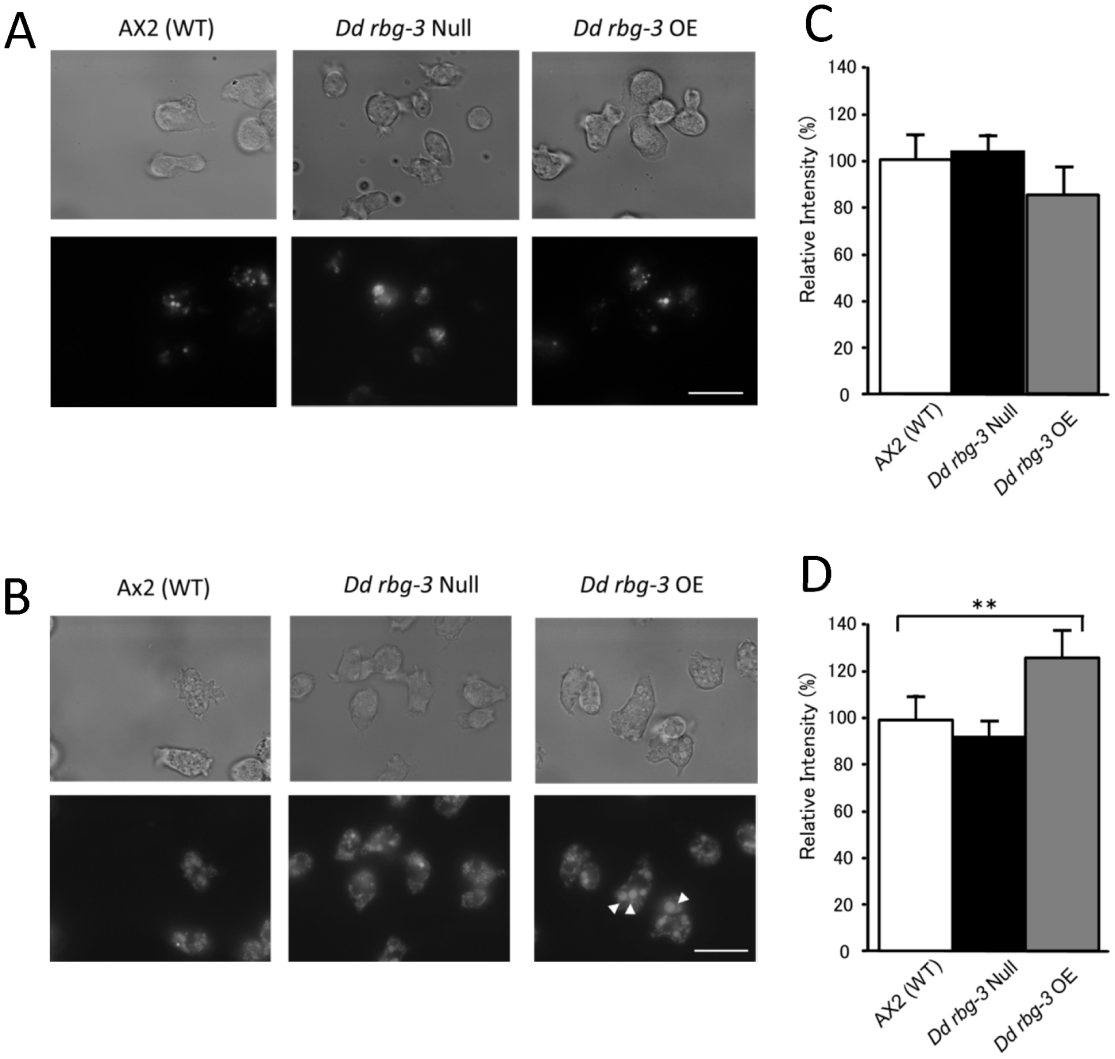
Endocytosis and lysosomes in AX2, *Dd rbg-3*-null, and *Dd rbg-3-*overexpression strains. (A) Fluorescence microscopy images of endocytosis of rhodamine-dextran. Vegetative cells were incubated with rhodamine-dextran [0.4 mg/ml] for 10 min, and then washed 3 times with PB. Scale bar, 10 µm. (B) Fluorescence microscopy images of lysosomes visualized using LysoTracker® Green. Vegetative cells were incubated with 75 nM LysoTracker® Green (Invitrogen, USA) for 30 min, and then washed 3 times with PB. Arrowheads indicate swollen lysosomes in the overexpression cells. Scale bar, 10 µm. (C) Relative fluorescent intensity of cell suspension treated with rhodamine-dextran [0.4 mg/ml] in 5×10^7^ cells/ml. (D) Relative fluorescent intensity of cell suspension stained with LysoTracker® Green in 5×10^7^ cells/ml. **P<0.05 between AX2 and *Dd rbg-3* OE (*t*-test).

During the growth phase, cells proliferate by internalizing fluid phase nutrients using endosomal machinery and pinocytosis, and thereafter traffic the nutrients to the lysosome for digestion. In *D. discoideum*, Rab has been implicated in the endosomal pathway during post-internalization steps [15]. To investigate how cell growth rate is affected in the Dd Rbg-3 mutants, intracellular endosomal and lysosomal structures were visualized using rhodamine-conjugated dextran and LysoTracker® Green, respectively. Endocytosis of liquid nutrients in neither mutant was significantly different from that in the parental strain, AX2 (Fig. 3A, 3C); however, the size of lysosomes in the overexpression mutant was larger than that in the parent strain (Fig. 3B, 3D). These data suggested that Dd Rbg-3 does not contribute to the control of the endocytotic pathway but is related to lysosomes.

### Dd Rbg-3 is involved in the regulation of adenylyl cyclase activity during development

**Figure 4:**
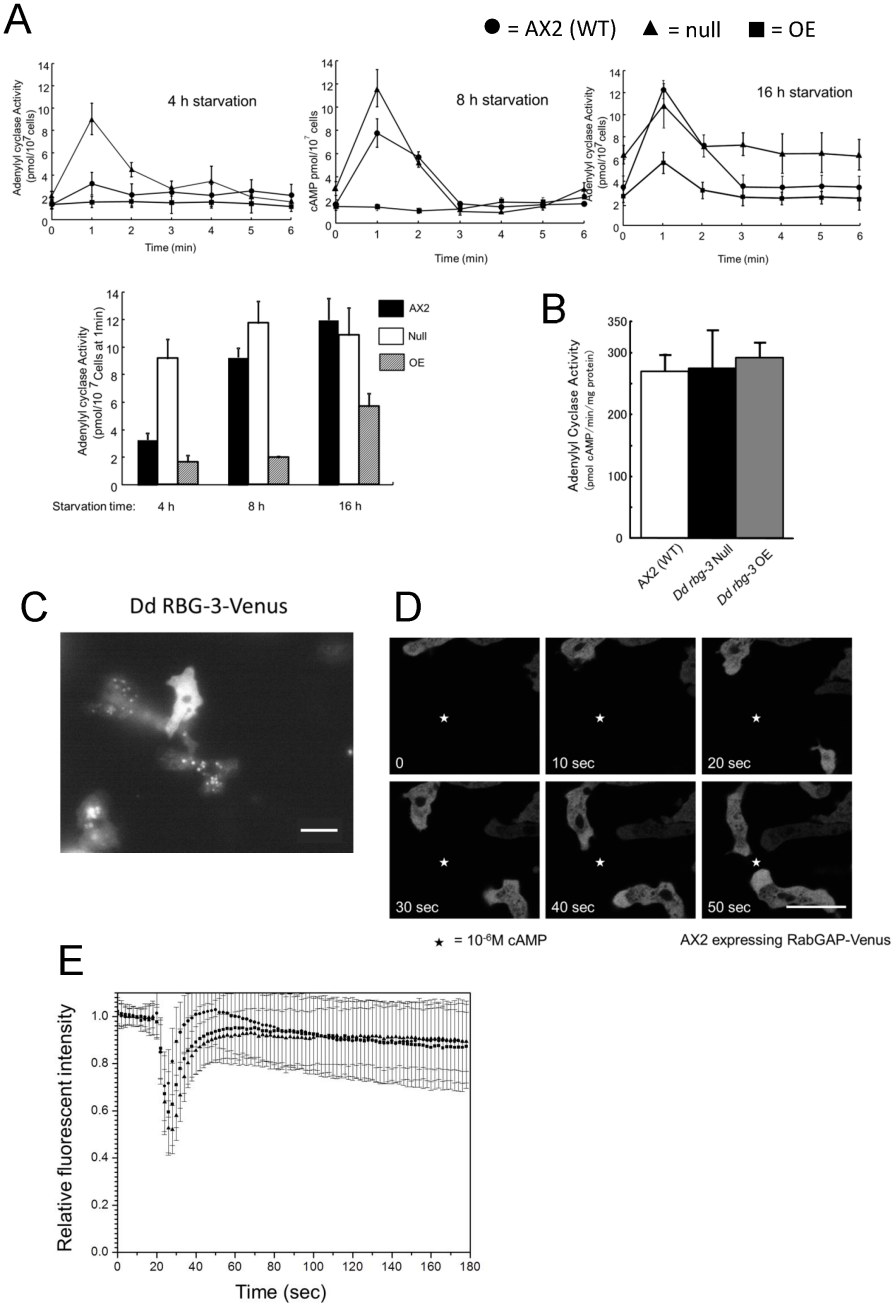
Adenylyl cyclase activity and cellular localization of Dd Rbg-3. (A) Adenylyl cyclase activity was measured in intact AX2 (filled circles, •), *Dd rbg-3*-null (filled triangles, ▴), and *Dd rbg-3*-overexpression (filled squares, ▪) cells following cAMP stimulation. Starved cells were stimulated with 10 µM deoxy-cAMP. At specific time-points, aliquots of cells were lysed and the cAMP levels measured. (B) Adenylyl cyclase activity in cell lysates of the parental and mutant strains measured in the presence of 30 µM GTPγS. (C) Fluorescent microscopy image of AX2 cells expressing Dd Rbg-3-Venus. Scale bar, 10 µm. (D) Fluorescent microscopy images of migration of Dd Rbg-3-Venus in AX2 cells toward a micropipette releasing 1 µM cAMP. Stars represent the position of the tip of the micropipette. Scale bar, 10 µm. (E) Translocation of CRAC. Aggregating cells were pretreated with 5 µM latrunculin A. Fluorescence intensity of CRAC-GFP in the cytosol was measured at the indicated time after stimulation with 1 µM cAMP. Cells were excited at 440 nm and viewed through a cut-off filter of 500–550 nm to assess emission before and after cAMP stimulation. In each experiment, fluorescence intensity of an area in the cytosol was measured at the indicated time-point and was normalized to the average intensity measured 18 s before cAMP stimulation (n = 102 for AX2, n = 102 for the null mutant, n = 143 for the overexpression mutant).

Periodical production and secretion of cAMP in response to extracellular cAMP plays a central role in development, since cells lacking adenylyl cyclase A [ACA] are incapable of forming multicellular structures [16]. In order to gain insight into the mechanisms by which the developmental process is respectively enhanced or delayed in *Dd rbg-3-*null or *Dd rbg-3*-overexpression mutants, we measured the adenylyl cyclase activity in these mutants during development. At the aggregation stage, 8 h after commencing starvation, the null cells showed enhanced cAMP levels compared to AX2; the cAMP levels characteristically increased rapidly upon activation of the cAMP receptor by the cAMP agonist, 2′-deoxyadenosine-3′,5′-cyclic monophosphate (dcAMP). This was followed by a steady decrease to basal levels within 6 min. In contrast, no increase in cAMP levels was observed in the overexpression mutant strain (Fig. 4A). At an earlier stage of development, at 4 h after commencing starvation, only the null mutant showed a significant increase in cAMP levels (Fig. 4A). The overexpression cells showed significant cAMP production only 16 h after commencing starvation (Fig. 4A).

To determine whether the mutant cells have enhanced or reduced adenylyl cyclase activity, the intrinsic adenylyl cyclase activity was measured for both mutants, using a cell-free system and the potent, non-hydrolysable G-protein activator, guanosine 5′-O-[gamma-thio]triphosphate (GTPγS) [17]. The activities of adenylyl cyclase in *Dd rbg-3–*null and *Dd rbg-3*–overexpression mutants were similar to that of AX2 (Fig. 4B). These results indicated that the aberrant cAMP responses in the null and overexpression mutants are not due to an intrinsic aberrant adenylyl cyclase activity, but are instead due to alterations in the regulation of adenylyl cyclase. Thus, Dd Rbg-3 is involved in a negative regulatory pathway for adenylyl cyclase.

Dd Rbg-3 was visualized in living cells by fusing Venus, a modified eYFP fluorescent protein, to the N-terminus of Dd Rbg-3. As can be seen in Fig. 4C, in cells that were expressing relatively low levels of Dd Rbg-3–Venus, the protein was clearly localized to intracellular vesicles. In cells expressing high levels of Dd Rbg-3–Venus, the protein was observed uniformly throughout the cytosol. When these cells were placed in a gradient of the chemoattractant cAMP, Dd Rbg-3–Venus was observed to be transiently localized inside of the leading edge (Fig. 4D). These results suggest that Dd Rbg-3 may function within the pseudopods upon chemotactic stimulation.

Since translocation of the cytosolic regulator of adenylyl cyclase (CRAC) to the plasma membrane is a prerequisite for the activation of adenylyl cyclase, we next studied the role of Dd Rbg-3 in CRAC translocation in the mutants [18], [19]. The kinetics of CRAC translocation from the cytosol to the plasma membrane in the mutant strains was measured after cAMP stimulation using a CRAC-GFP fusion-protein. Upon stimulation with cAMP, GFP fluorescence in the cytosol region was transiently decreased in the parental strain and both mutant strains (Fig. 4E), with similar kinetics. Therefore, we deduced that Dd Rbg-3 is not involved in activating the translocation of CRAC.

### GAP activity of Dd Rbg-3 is indispensable for life-span regulation

**Figure 5:**
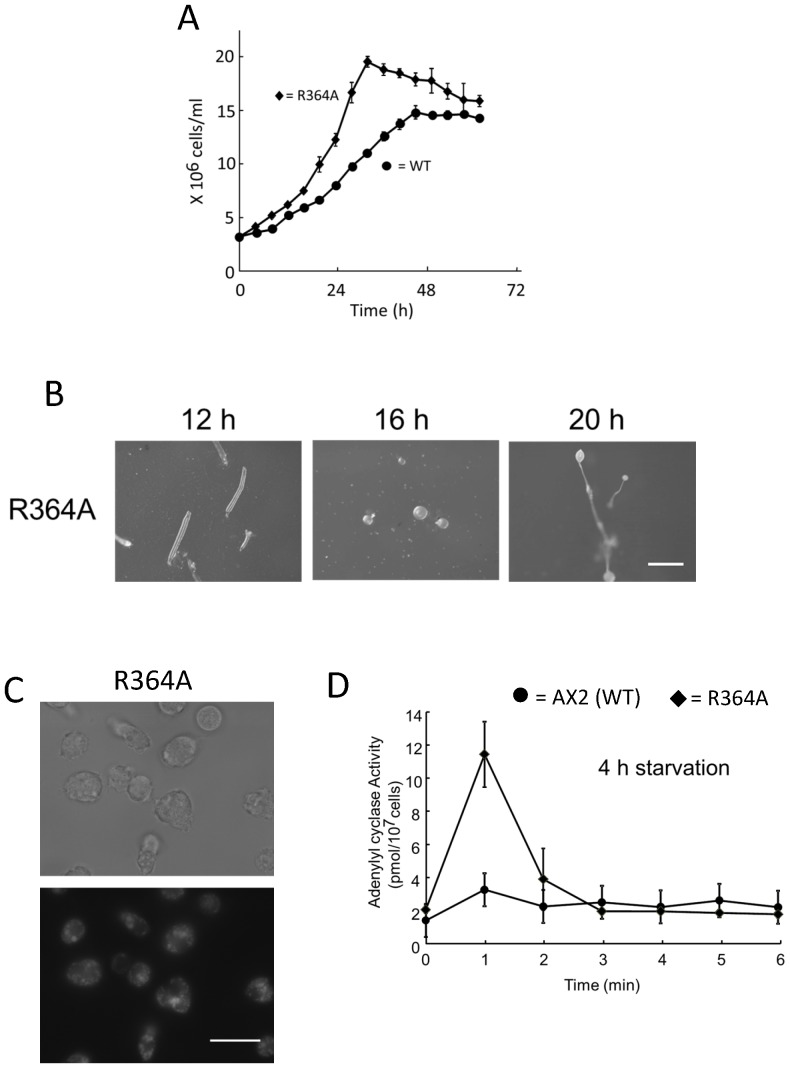
Phenotype of a R364A mutant in *Dd rbg-3*–null cells. (A) Cell growth in AX2 (filled circles, •) and the R364A (open triangles, △) strains. Cells were cultured in HL5 liquid medium, at 21°C, with shaking at 125 rpm. (B) Development in the R364A was assessed on non-nutrient agar plates; cells were plated at a density of 1×10^6^ cells/cm^2^. Scale bar, 1 mm.(C) Fluorescence microscopy images of lysosomes in AX2 and the R364A cells using LysoTracker® Green. Scale bar, 10 µm. (D) Adenylyl cyclase activity was measured in intact AX2 (filled circles, •) and R364A (filled diamonds, ♦) cells starved for 4 h following cAMP stimulation. Starved cells for 4 h were stimulated with 10 µM deoxy-cAMP.

To investigate whether the GAP activity of Dd Rbg-3 is involved in the life-span regulation, a mutated *Dd rbg*, R364A, which lacks GAP activity, was generated by a single amino acid substitution and constitutively expressed in the *Dd rbg-*3-null mutant^9^. The expression resulted in no distinct complementation on the life-cycle duration, the lysosomal morphology, the adenylyl cyclase activities, indicating that the GAP activity is involved in life-span regulation (Fig. 5).

### Rab7A is a potential target of Dd Rbg-3

**Figure 6:**
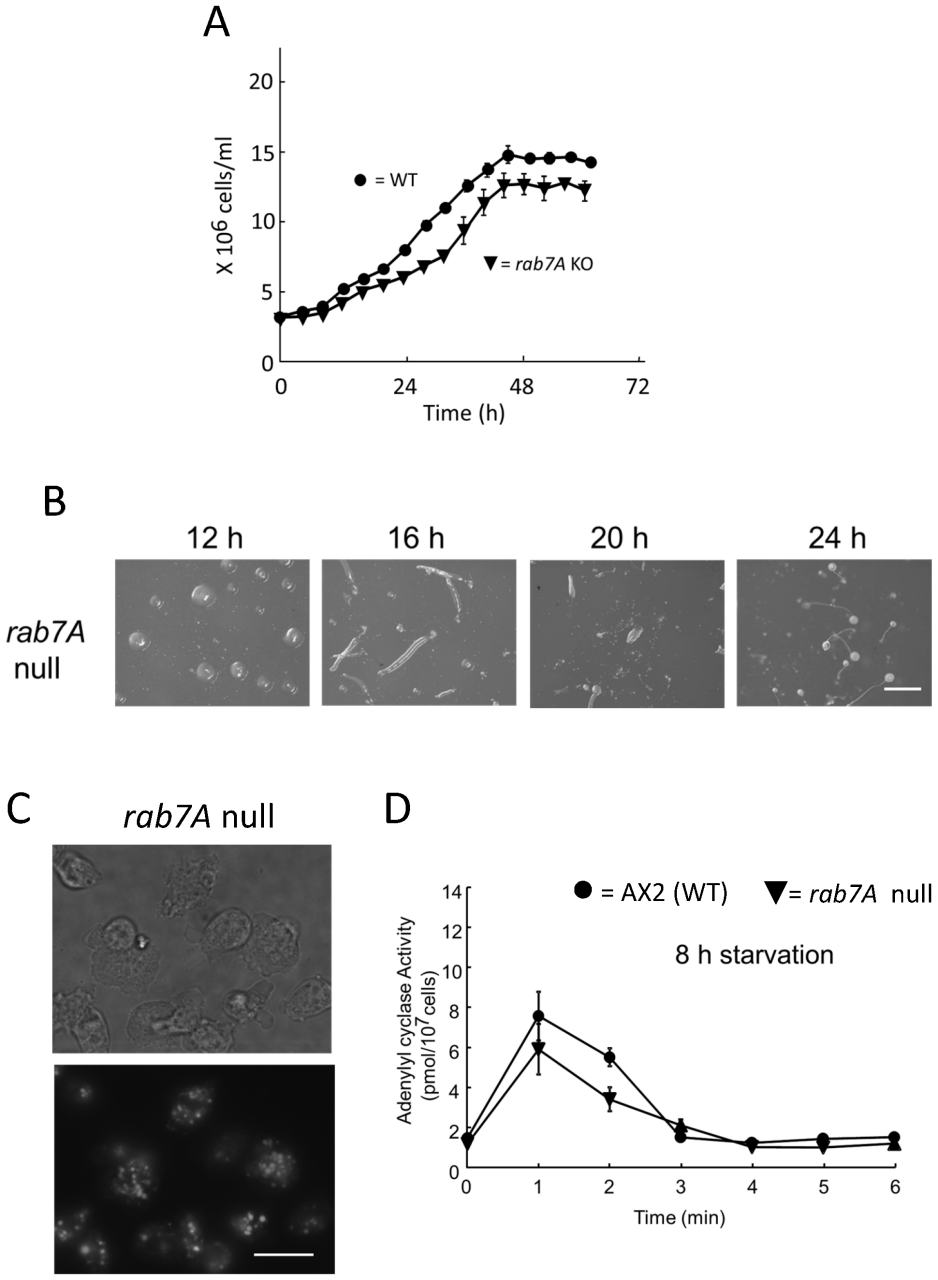
Phenotype of *rab*7A–null mutant. (A) Cell growth in AX2 (filled circles, •) and the rab7A–null (open triangles, △) strains. Cells were cultured in HL5 liquid medium, at 21°C, with shaking at 125 rpm. (B) Development in rab7A-null was assessed on non-nutrient agar plates; cells were plated at a density of 1×106 cells/cm^2^. Scale bar, 1 mm. (C) Fluorescence microscopy images of lysosomes in AX2 and the *rab7A*-null cells using LysoTracker® Green. Scale bar, 10 µm. (D) Adenylyl cyclase activity was measured in intact AX2 (filled circles, •) and *rab7A*-null (inverted filled triangle, ▾) cells starved for 8 h following cAMP stimulation. Starved cells for 4 h were stimulated with 10 µM deoxy-cAMP.

Because Rbg-3 of *C. elegans* and its homologue in humans, TBC1D5, were shown to possess GAP activities for rab7, Dd Rbg-3 is also likely to function as a GAP for the *D. discoideum* rab7 homologue, rab7A. The *rab7A*–null mutant generated showed slow growth and increased number of but did not exhibit a delayed developmental time course and like the *Dd-rbg3*-overexpression mutant, suggesting that rab7A is a potential target of Dd-Rbg3 in growth phase (Fig. 6).

### Dd Rbg-3 is potentially regulated via trimeric G-proteins

**Figure 7:**
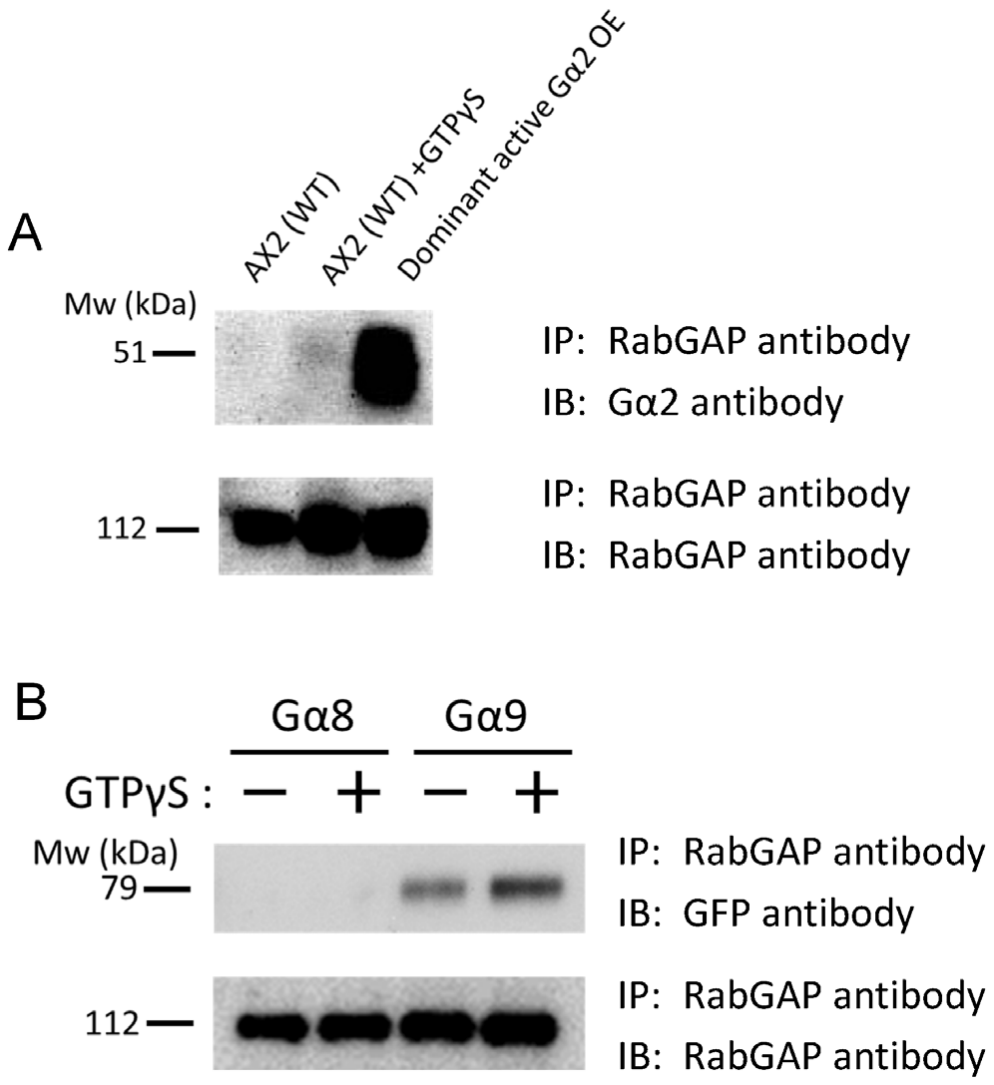
*In vivo* interaction of Dd Rbg-3 with activated Gα2 and Gα9. (A) Co-immunoprecipitation of Gα2 with Dd Rbg-3. Lysates of AX2 cells and cells overexpressing dominant gα2 were used. (B) Co-immunoprecipitation of Gα8 and Gα9 with Dd Rbg-3. Lysates of AX2 cells and cells expressing eGFP-fused Gα8 and Gα9 were used. In each experiment, cell lysates were incubated with or without 30 µM GTPγS before the pull-down experiments.

Given that Dd Rbg-3 had been isolated as a putative binding partner of activated Gα2, we next examined this interaction *in vivo* by pull-down assay using specific antibodies against Gα2 and Dd Rbg-3. In cell lysates incubated with GTPγS, Dd Rbg-3 was co-immunoprecipitated, whereas little Dd Rbg-3 could be co-immunoprecipitated when cells were not exposed to GTPγS (Fig. 7A). Furthermore, this co-immunoprecipitation was increased in cell lysates prepared from cell lines expressing dominant Gα2 (Fig. 7A). Thus, these results suggested that Dd Rbg-3 is regulated via a Gα2-dependent pathway, by interaction with the activated form of Gα2.

Previously, it was reported that cells lacking Gα8 and Gα9 grow faster than wild-type cells [20]. To examine whether these Gα subunits also interact with Dd Rbg-3 during the growth phase, we expressed the C-terminal GFP-fused G proteins in wild-type cells and performed the pull-down assay with the anti-Dd Rbg-3 antibody with or without GTPγS. While little Gα8 was co-immunoprecipitated with anti-Dd Rbg-3 and no enhancement was observed by GTPγS, a significant amount of Gα9 was precipitated and this was enhanced by GTPγS (Fig. 7B). Together, these observations suggest that Dd Rbg-3 is potentially regulated via the activity of Gα9 during the growth phase and that of Gα2 during the developmental phase.

### Human Rbg-3 complements the null phenotype

**Figure 8:**
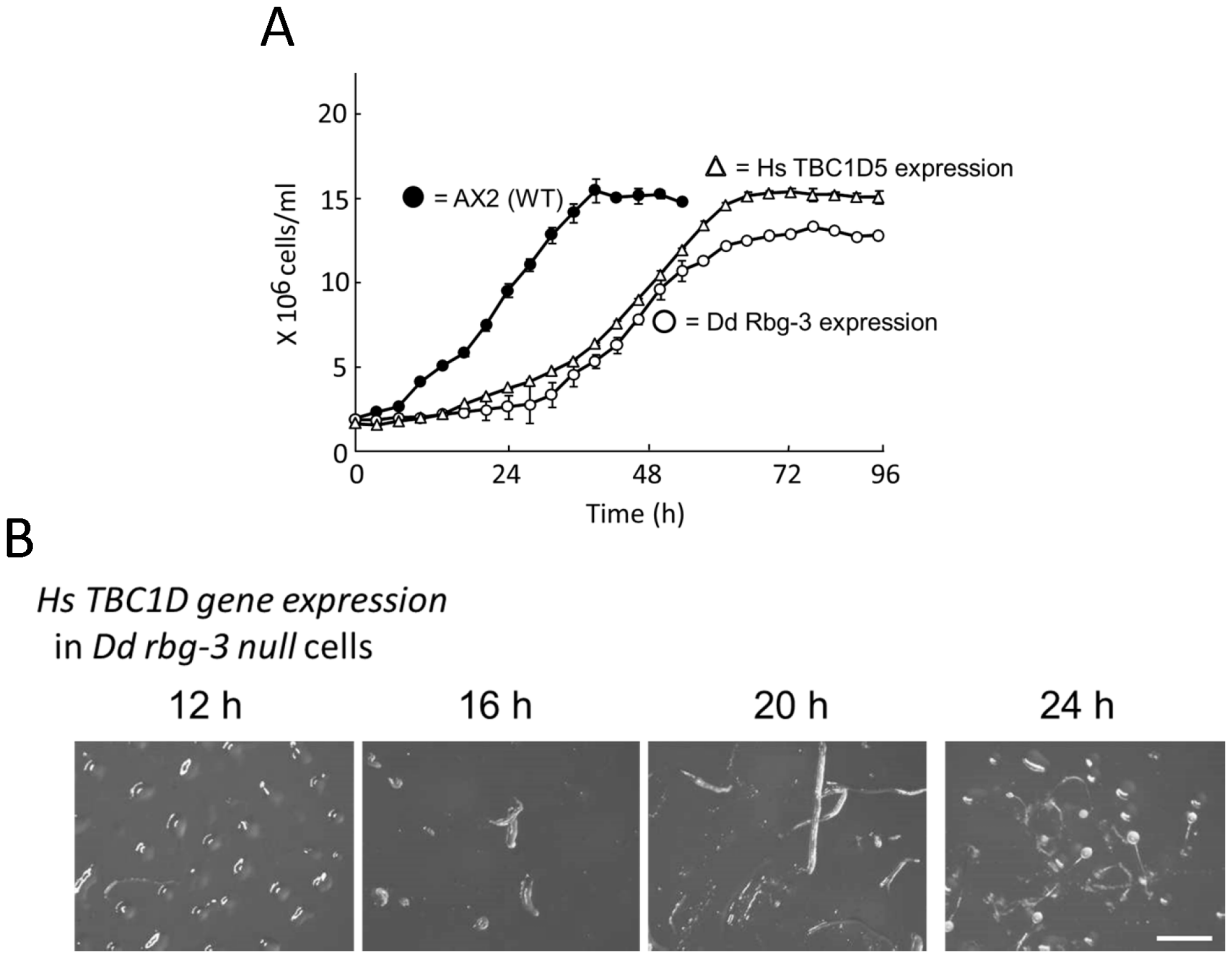
Complementation of *Dd rbg-3–null* cells by expressing human *Rbg-3* homolog. (A) Growth in AX2 cells (filled circles, •), Dd rbg-3-null cells (open circles, ○), and Dd rbg-3-null cells expressing the human homologue, TBC1D5 gene (open triangles, △), cultured in HL5 liquid medium with shaking. (B) Development of Dd rbg-3-null cells expressing human *Rbg-3* incubated on non-nutrient agar plates, at a density of 1×10^6^ cells/cm^2^. Scale bar, 1 mm.

Phylogenic analysis revealed that *rbg-3* is a gene widely conserved across species, from the slime mold to *Homo sapiens* (Fig. S3). We next investigated whether expression of the human Rbg-3 homolog could rescue the phenotype of the null mutation in *D. discoideum*. Cells expressing the full-length human *rbg-3, tbc1D* cDNA complemented rapid cell growth and precocious development (Fig. 8A and 8B). Furthermore, a *C. elegans rbg-3* homolog could partially, but significantly, rescue this null phenotype (Fig. S4). These results indicated that both mammalian and nematode Rbg-3 are functional in *D. discoideum*, suggesting that Rbg-3 may fulfill the same general function in those organisms.

### *Dd rbg-3*-null mutant is resistant to caffeine, an inhibitor of adenylyl cyclase

**Figure 9:**
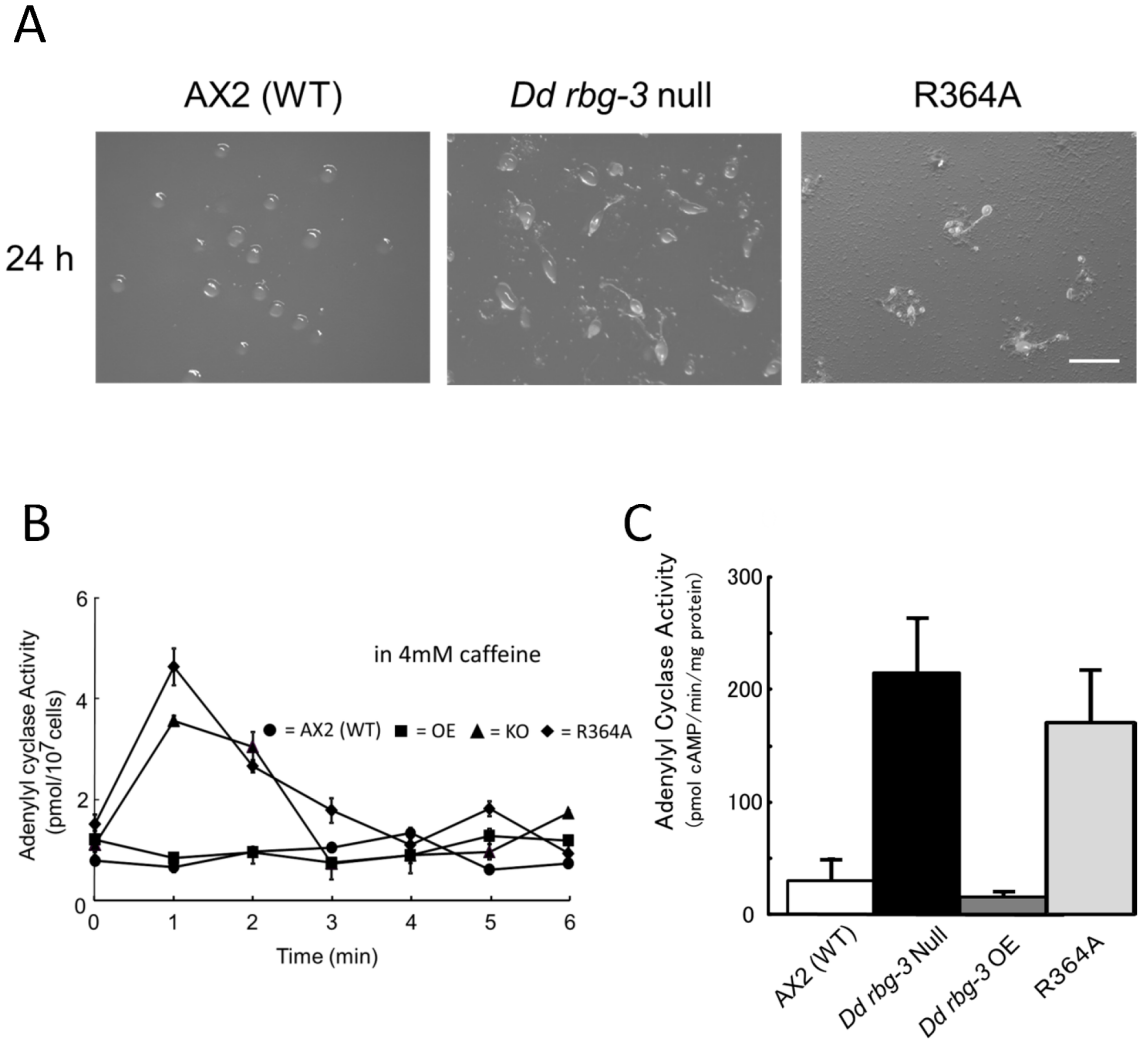
Caffeine does not inhibit the development, or adenylyl cyclase activity, of *Dd rbg-3*-null mutant cells and cells expressing the R364A-mutant *Dd rbg-3*. (A) Development of AX2 and *Dd rbg-3-null* cells starved at a density of 1×10^6^ cells/cm^2^ on non-nutrient agar plates containing 4 mM caffeine. In the parental cells, slugs and fruiting bodies were not observed. Scale bar, 10 µm. (B) Adenylyl cyclase activity was measured, following stimulation with 10 µM deoxy-cAMP, in intact cells grown under starvation conditions for 8 h in the presence of 4 mM caffeine. (C) Adenylyl cyclase activity was measured in the presence of 30 µM GTPγS, in cell lysates grown in medium containing 4 mM caffeine.

Caffeine is known to specifically constrain cAMP accumulation by inhibiting adenylyl cyclase in *D. discoideum*, although the exact molecular mechanisms involved are unclear [17]. As the *Dd rbg-3-*null and R364A mutants exhibited an enhanced developmental phenotype and cAMP production, we tested whether the mutant was affected by caffeine. Interestingly, the null mutant could form a normal fruiting body by 24 h, whereas the parental cells could not develop further on the non-nutrient agar surface containing 4 mM caffeine (Fig. 9A).

Next, we examined adenylyl cyclase activity directly in these strains in the presence and absence of 4 mM caffeine. Intact cells were stimulated with dcAMP in the presence of 4 mM caffeine. While neither the parental cells nor the overexpression mutants showed any production of cAMP (Fig. 9B), the null and R364A cells transiently accumulated a small but significant amount of cAMP (Fig. 9B). The resistance of adenylyl cyclase activity in the null and R364A mutants to caffeine were also observed in a cell-free system (Fig. 9C). These results indicated that Dd Rbg-3 GAP activity is involved in the inhibition of adenylyl cyclase by caffeine.

## Discussion

In this study, we identified Dd Rbg-3 as a temporal life-cycle regulator in *Dictyostelium*; absence of the protein resulted in fast proliferation and accelerated development of the organism, whereas the opposite effect was observed when the protein was overexpressed.

In *Dictyostelium*, adenylyl cyclase activity has been shown to play a central role in development, acting as an intracellular second messenger and as an extracellular chemoattractant [21]. When adenylyl cyclase activity is lacking, cells do not develop under starving conditions. However, overexpression of the catalytic subunit of cAMP-dependent protein kinase [PKA], the main intracellular cAMP signaling receptor, or deletion of the PKA regulatory subunit, which inhibits the catalytic activity of PKA, causes a rapid development phenotype, albeit with an aberrant fruiting body typical of a sporogenous mutant [22], [23]. We measured *in vivo* adenylyl cyclase activity in the null and overexpression mutants, which exhibited precocious or serotinous development, respectively. We showed that the null mutant of Dd *Rbg-3* does not exhibit the precocious differentiation and sporogenous phenotype; thus, deletion of *Dd Rbg-3* does not seem to cause hyperactivation of cAMP signaling, but instead appears to regulate precocious activation of adenylyl cyclase during the course of development. This indicates that Dd Rbg-3 may have dual regulatory pathways, regulating both lysosomal and adenylyl cyclase signaling.

In the cytosol, ACA is found encapsulated in cytosolic vesicles and is carried to the plasma membrane region [24], [25]. Since *Dd Rbg-3* encodes a Rab GTPase activating protein, it seems likely that Dd Rbg-3 would participate in ACA-vesicle trafficking via specific Rab[s]. However, accumulation of ACA at the plasma membrane in the *Dd rbg-3*-null and *Dd rbg-3*-overexpression mutant cells were not different from that in the parental cells (Fig. S5). Furthermore, our findings indicate that CRAC translocation is not involved in the Dd Rbg-3 dependent cascade, since the transient translocation of CRAC following cAMP stimulation was clearly observed in the *Dd rbg-3*-null as well as the *Dd rbg-3*-overexpression mutant cells.

Although the mechanism by which Dd Rbg-3 regulates adenylyl cyclase is yet to be elucidated, we propose that Dd Rbg-3 is regulated via cAMP receptor-dependent Gα2 activation, because Dd Rbg-3 could be co-immunoprecipitated only with the active form of Gα2. Dd Rbg-3 contains a TBC domain that is responsible for interacting with Rab small G-proteins. Because Gα2 possesses a GTP hydrolysis activity similar to that of Rabs, the question arises whether the interaction of these proteins was fortuitous. The prey clone isolated by Y2H encoded only a C-terminal part of Dd-Rbg-3, which did not contain the TBC domain. Therefore, we consider that the co-immunoprecipitation between Dd Rbg-3 and Gα2 was not an artifact caused by non-specific binding of the TBC domain to the GTP-binding domain of Gα2.

Caffeine is known to inhibit ligand-induced adenylyl cyclase activation in *Dictyostelium* [26]. Caffeine inhibits all adenylyl cyclases, viz., ACA, ACB, and ACG in *Dictyostelium*, and although the exact molecular mechanism of action remains unclear, this inhibition is neither due to ceasing of the secretion of the cAMP produced nor due to hyperactivation of cAMP phosphodiesterases [17]. We found here that the *Dd Rbg-3*-null mutant exhibited a caffeine-resistant phenotype and was capable of producing cAMP under a physiologically effective caffeine concentration like cells lacking phospholipase A2 gene [27], indicating that caffeine acts on adenylyl cyclase via Dd Rbg-3. Although we have yet to determine whether caffeine acts directly on Dd Rbg-3 or via other components, our results indicate that Dd Rbg-3 is involved in the caffeine-mediated inhibition of adenylyl cyclase activity.

In *C. elegans*, RBG-3 was first identified as a novel interaction partner for TUB-1 [10]. Mutations in tubby are known to be related to the extension of life-span, partly via its interaction partner, Rbg-3, although the underlying mechanisms are unidentified [11]. In *D. discoideum*, it was revealed that Rbg-3 homologue is also found to be related to the life span and, furthermore, the signaling cascade was identified to involve both lysomal and cAMP signaling pathways, which suggests that a similar mechanism may possibly regulate the tubby- Rbg-3 cascade in *C. elegans*.

The TBC domain of Rbg-3 is significantly homologous to TBC1D5, which is known to negatively regulate Rab7 and, thus, to inhibit endosome-to-Golgi retrieval [28]. Identifying the component downstream of Rbg-3 would yield a key to unravel the signaling pathway. *C. elegans* RBG-3 was shown to preferentially stimulate the intrinsic activity of RAB7 in an *in vitro* mammalian system [12]. In *D. discoideum*, it was previously shown that Rab7A regulates both the early and late steps of phagosome maturation [28]. Activation of Dd Rbg-3 most likely transforms Rab7A from the active to the inactive state [29]. Furthermore, we found in this study that *rab*7A-null mutant partially mimics the phenotype of the overexpression mutant. Therefore, our observation that overexpression of Dd Rbg-3 results in swollen lysosomes is probably due to the maintenance of Rab7A in a constitutively inactive state. Thus, RabGAP most probably negatively regulates growth rate in the vegetative stage via Rab7A-dependent lysosomal trafficking.

There are lines of evidence indicating that growth rate is regulated via a G-protein-coupled receptor in *D. discoideum*. A deletion mutant of the G-protein-coupled receptor encoded by *crlA* grows to a higher cell density, similar to the *Dd rbg-3*-overexpression mutant [30]. It could be hypothesized that a Gα protein regulates Dd Rbg-3 during cell proliferation; Gα8 and Gα9 are such candidates, since deletions of the corresponding genes also result in faster proliferation [31]. Since Gα9 interacts with Dd Rbg-3, it is suggested that Gα9 may participate in the regulatory pathway for Dd Rbg-3 during vegetative phase and may play a role in cell proliferation.

Several effector proteins that directly bind Gα subunits have been isolated to date [32]. Plasma membrane-bound adenylyl cyclase is such a protein, and it is activated by direct binding to the activated form of Gαs in other cell types. Considering that *Dd Rbg-3* was isolated by Y2H library screening with Gα2; that the interaction was confirmed by co-immunoprecipitation in a cytosolic fraction, which does not contain ACA; and that ACA is not directly activated by Gα2 in *D. discoideum* cells [33], it seems reasonable to suppose that Dd Rbg-3 is regulated via the activated form of Gα2 leading to ACA signaling. Further experiments are required to evident this hypothesis.

We showed that the ubiquitously distributed Dd Rbg-3 transiently translocates to the inside of the leading pseudopods in chemotaxing cells. However, the null mutant cells aggregated normally under starvation, and chemotaxis activity toward extracellular cAMP was also fairly normal. Therefore, Dd Rbg-3 appears to only play a role in a cAMP signaling pathway that is not related to the chemotactic signaling pathway.

Rbg-3 homologs are present in divergent multicellular organisms but are not found in unicellular organisms, like yeast (Fig. S3). In this study, we showed that human as well as nematode Rbg-3 complemented the *in vivo* function of Dd Rbg-3, indicating that a common functional role may exist for Rbg-3 among multicellular organisms. Further elucidation of a Gα cascade involving the Rbg-3 pathway in other multicellular organisms would be required to unravel the common and divergent functions of this protein in different species.

## Materials and Methods

### *Dictyostelium* Cell Culture and preparation for Development and adenylyl cyclase analyses

Wild-type *Dictyostelium discoideum* AX2 cells were cultured at 21°C, with shaking at 125 rpm, in HL5 medium containing 100 µg/ml streptomycin sulfate and 100 units/ml benzylpenicillin potassium [34]. For culturing the transformed cells used in this study, HL5 medium was supplemented with the selecting drugs, viz., 10 µg/ml blasticidin S or 20 µg/ml G418. Cells were collected by centrifugation and resuspended in PB (10 mM Na_2_HPO_4_ and 10 mM KH_2_PO_4_ [pH 6.5]).

For analysis of development, AX2 and mutant cells were juxtaposed on a 1.5% agar PB plate or a nitrocellulose filter placed on top of the agar plates, at a surface density of 1×10^6^ cells/cm^2^. For the adenylyl cyclase assay, cells were suspended in PB at a density of 1×10^7^ cells/ml and shake-cultured at 125 rpm for 1 h; thereafter, 30 nM cAMP was added in repeated pulses every 6 min [35]. Then, cell density was adjusted at a density of 1×10^8^ cells/ml.

### Vector construction and transformation

A gene-targeting construct for generating a *Dd Rbg-3*–null and *rab7A*–null mutant was prepared by inserting the blasticidin S resistance gene expression cassette (*bsr*) into the coding region using the fusion PCR technique [34], [36], [37]. A gene-targeting constructs for generating *Dd Rbg-3*–null and rab7A-null mutants were prepared by inserting the blasticidin S resistance gene expression cassette (*bsr*) into the coding region using the fusion PCR technique. The linear construct was amplified by PCR. The full-length *Dd Rbg-3* was amplified from a cDNA mixture prepared from aggregation-stage mRNA using an oligo-dT primer and was verified by sequencing. Full length clones of human and *C. elegans Rbg-3* genes were provided by NBRC (FLJ No: FLJ17581) and by Dr. Kohara (clone ID: yk1627a12), respectively. For constructing the overexpression and fluorescent protein fusion vectors, the full-length *Dd Rbg-3* cDNA was cloned into the cloning site of pHK12neo or pHK12neo-N-Venus. The cDNA of the dominant form of Gα2 (Q208L) and the dominant negative form of *Dd* Rbg-3 (R364A) was generated using a Multi Site-directed Mutagenesis kit (MBL, Japan) and the following DNA oligomer: 5′-CTTTCTTTCAGAACGTAAACCACCAACATCTAC-3′ and. CACAAAAAATTATTAAAATTGATTTAGAGGCAACTCATCCAGATAATG-3′, respectively. The expression vectors for *gα8* and *gα9* genes fused with eGFP at C-terminal regions were made by cloning into pHK12neo-C-eGFP. Transformation by electroporation was performed using 10 µg of the generated DNA fragment or vectors; this and selection of transformants were accomplished as described previously.

### Nucleic acid analyses

Total RNA was extracted using an RNAeasy kit (Qiagen, Germany). Genomic DNA was extracted as described before. Blotting and detection for northern and Southern analyses were performed using the DIG Easy Hyb kit (Roche Diagnostics K. K., Germany).

### Adenylyl cyclase and chemotaxis assays

Adenylyl cyclase activity was assayed as previously described [17], [37]. Chemotaxis assays were performed as described before [38].

### Fluorescence microscopy and measurement

Vegetative and differentiated cells were prepared as described above. Cells were viewed using a Zeiss Axiovert 200 M fluorescence microscope (Carl Zeiss, Germany). CRAC translocation and ACA localization was measured in aggregating cells expressing CRAC-GFP or ACA-eYFP, using an A1 confocal laser microscope system (Nikon Instruments Inc., Japan).

Fluorescent intensities of cells were measured with Spectrofluorophotometer RF-5300PC (Shimadzu Japan). Cells were stained with 75 nM LysoTracker® Green and resupended at a density of 5×10^7^ cells/ml. The fluorescent intensities were measured with 500 nm as excitation and 520 nm as emission.

### Antisera and protein analyses

Anti-Dd Rbg-3 and Gα2 sera were raised in rabbits against an N-terminal polypeptide region [DINSGTTTPNIRNS] of Dd Rbg-3, and a C-terminal polypeptide region of Gα2 [CVMKAGLYS], respectively; these antibodies were affinity purified to each immunized polypeptide. These antipeptide sera were produced by Scrum Inc., Japan. Western blot analyses were performed using total protein extracted from aggregating cells, following a standard protocol supplied by ATTO Corporation, Japan. Co-immunoprecipitation was performed using a Dynabeads® Co-Immunoprecipitation kit (Invitrogen Corporation, CA, USA) following the manufacturer's protocol.

## Supporting Information

Figure S1 **Confirmation of** ***Dd Rbg-3*** **transformants by southern and northern analyses.** (A) Schematic diagram of the *Dd rbg-3* gene targeting construct. (B) Southern blot analysis of the *Dd rbg-3*-null mutant. (C) Northern blot analysis of the *Dd rbg-3* [*Dd rbg-3*-Null]-null and -overexpression (*Dd rbg-3* OE) mutants.
(TIF)

Figure S2 **Northern blot analyses monitoring expression of developmental genes in** ***Dd rbg-3*** **-null and** ***Dd rbg-3*** **-OE mutants.** These blots indicate that both *carA* and *gpaB* are expressed early in development. Expression of *ecmA* and *pspA* indicate that they are prestalk and prespore genes, respectively.
(TIF)

Figure S3 **Phylogenic analysis of** ***Rbg-3*** **.** The phylogenetic tree shown was constructed from amino acid sequences of *Rbg-3* homologues from the various species.
(TIF)

Figure S4 **Complementation of** ***Dd Rbg-3-null*** **cells by expressing** ***C. elegans Rbg-3*** **homolog.** Development of *Dd rbg-3*-null cells expressing *Caenorhabditis elegans Rbg-3* grown on non-nutrient agar plates, at a density of 1×10^6^ cells/cm^2^. Scale bar: 1 mm.
(TIF)

Figure S5 **Cellular localization of adenylate cyclase A (ACA).** Fluorescent microscopy images of ACA-eYFP in AX2, *Dd Rbg-3*-null and -over-expression (OE) cells. Scale bar: 10 µm.
(TIF)
