# Supplementary figures and images for: A RabGAP Regulates Life-Cycle Duration via Trimeric G-protein Cascades in Dictyostelium discoideum
Source: PLoS One. 2013 Dec 11;8(12):e81811. doi: 10.1371/journal.pone.0081811 (PMC3859538; doi:10.1371/journal.pone.0081811)

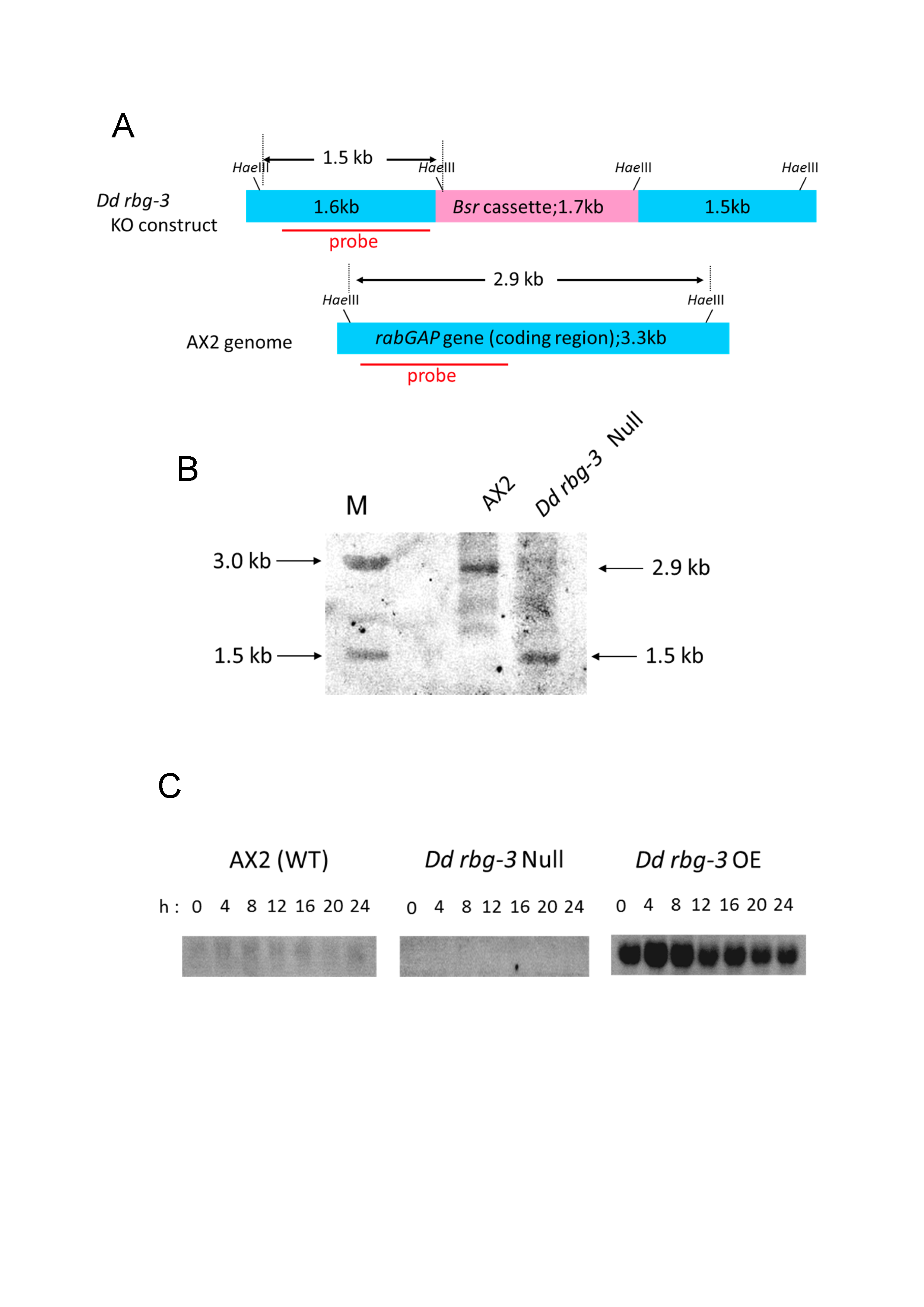

Supplement: Figure S1 — Confirmation of Dd Rbg-3 transformants by southern and northern analyses. (A) Schematic diagram of the Dd rbg-3 gene targeting construct. (B) Southern blot analysis of the Dd rbg-3-null mutant. (C) Northern blot analysis of the Dd rbg-3 [Dd rbg-3-Null]-null and -overexpression (Dd rbg-3 OE) mutants. (TIF) [file pone.0081811.s001.tif]

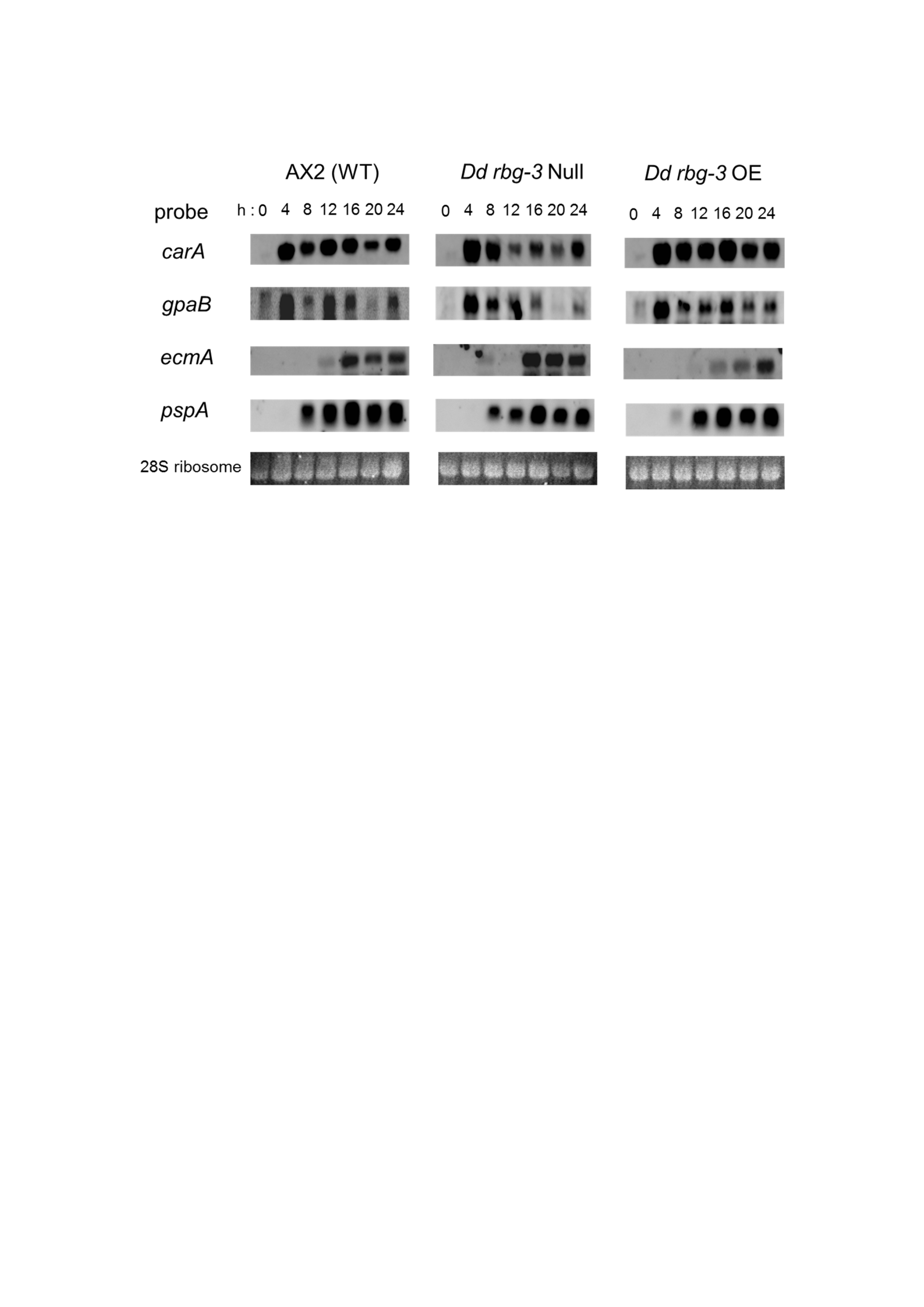

Supplement: Figure S2 — Northern blot analyses monitoring expression of developmental genes in Dd rbg-3 -null and Dd rbg-3 -OE mutants. These blots indicate that both carA and gpaB are expressed early in development. Expression of ecmA and pspA indicate that they are prestalk and prespore genes, respectively. (TIF) [file pone.0081811.s002.tif]

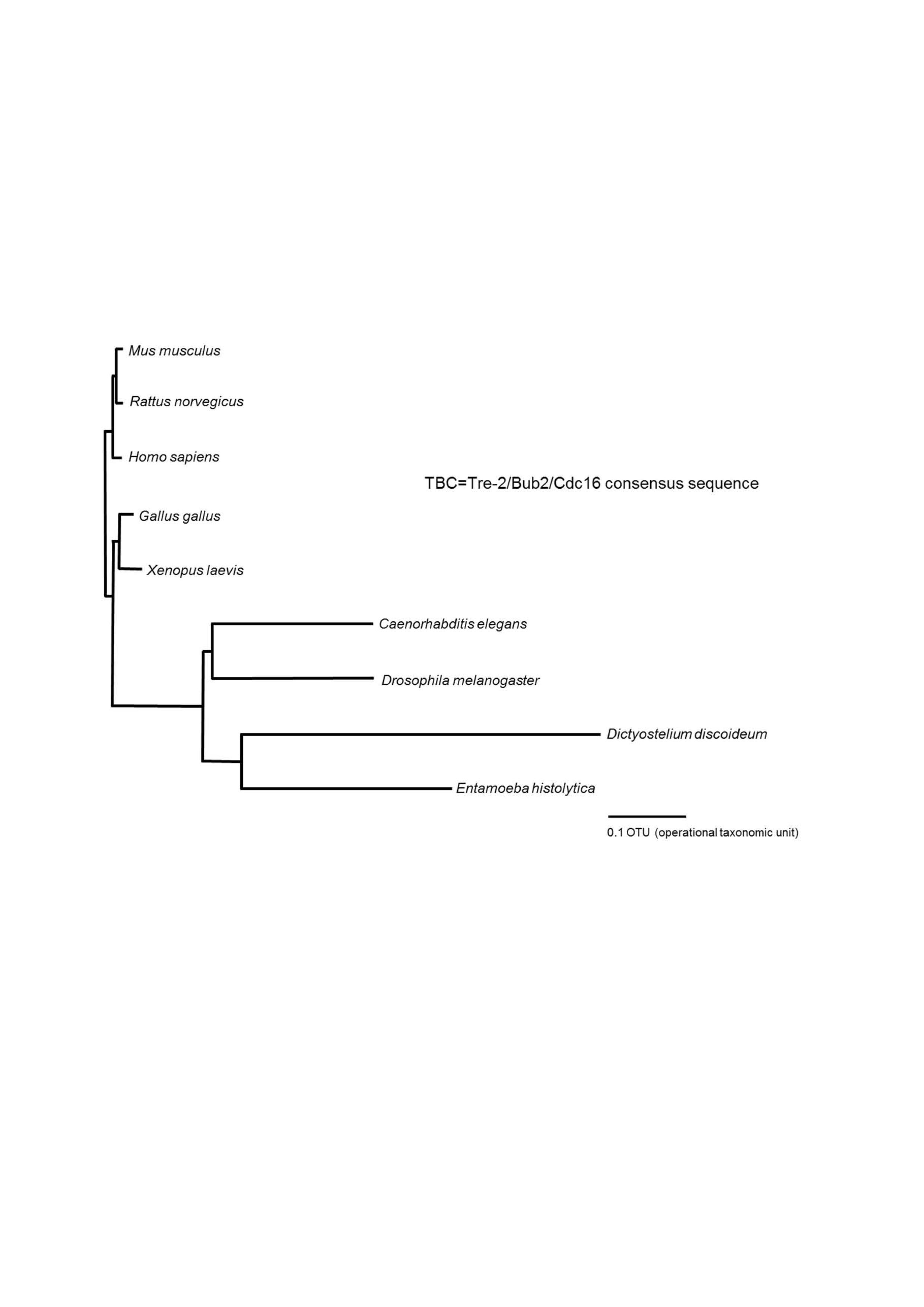

Supplement: Figure S3 — Phylogenic analysis of Rbg-3 . The phylogenetic tree shown was constructed from amino acid sequences of Rbg-3 homologues from the various species. (TIF) [file pone.0081811.s003.tif]

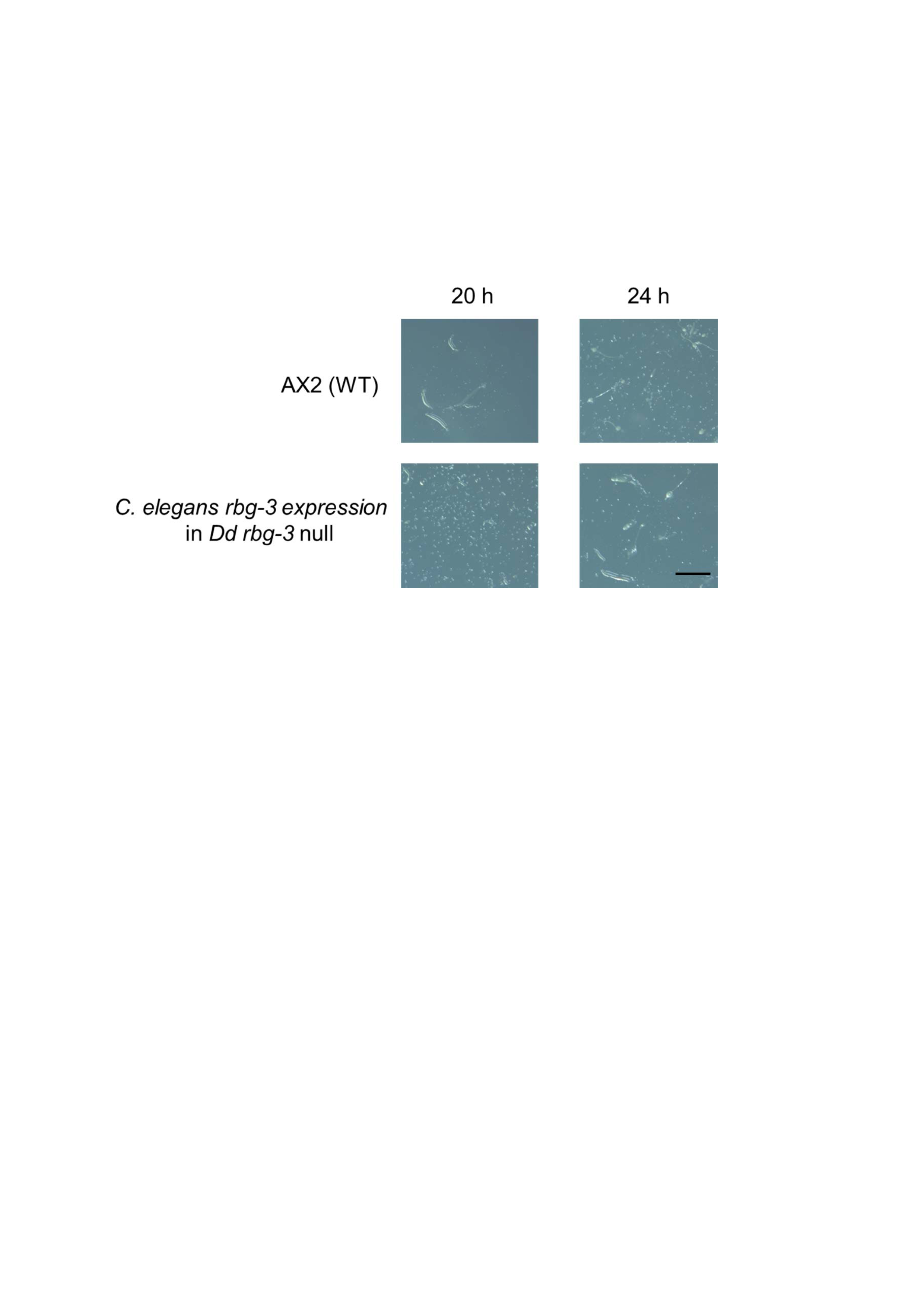

Supplement: Figure S4 — Complementation of Dd Rbg-3-null cells by expressing C. elegans Rbg-3 homolog. Development of Dd rbg-3-null cells expressing Caenorhabditis elegans Rbg-3 grown on non-nutrient agar plates, at a density of 1×10^6^ cells/cm2. Scale bar: 1 mm. (TIF) [file pone.0081811.s004.tif]

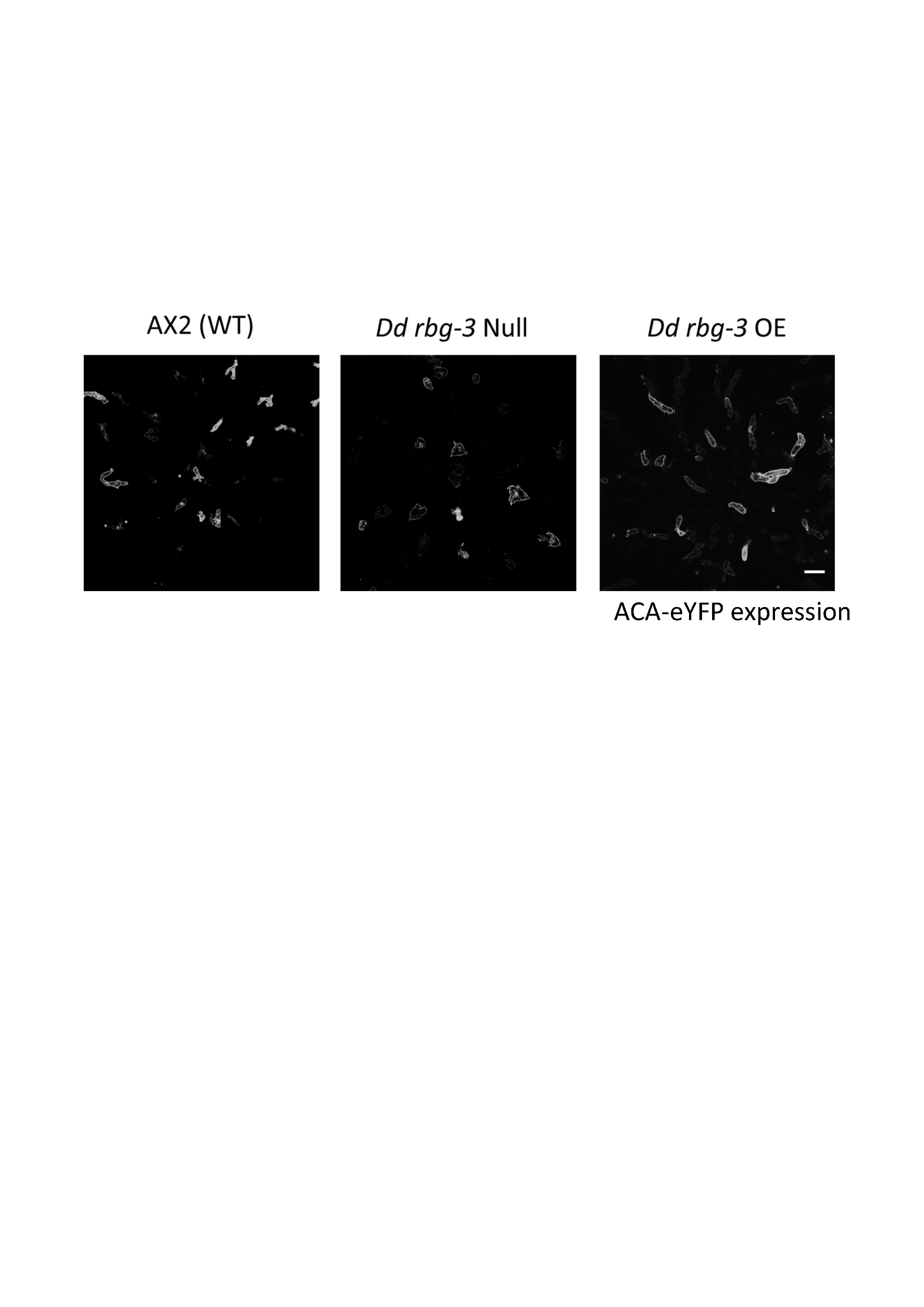

Supplement: Figure S5 — Cellular localization of adenylate cyclase A (ACA). Fluorescent microscopy images of ACA-eYFP in AX2, Dd Rbg-3-null and -over-expression (OE) cells. Scale bar: 10 µm. (TIF) [file pone.0081811.s005.tif]
